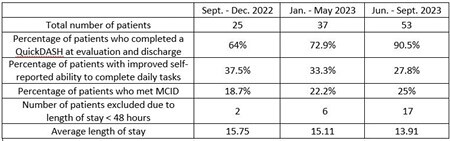# 117 QuickDASH Quantifies Perceived Upper Extremity Function in Acute Burn Rehabilitation: A Quality Improvement Project

**DOI:** 10.1093/jbcr/irae036.116

**Published:** 2024-04-17

**Authors:** Katie Coakley, AnnMarie Berry, Jaime Serrano

**Affiliations:** Loyola University Medical Center, Chicago, IL; Loyola University Medical Center, Maywood, IL; Loyola University Medical Center, Lombard, Illinois; Loyola University Medical Center, Chicago, IL; Loyola University Medical Center, Maywood, IL; Loyola University Medical Center, Lombard, Illinois; Loyola University Medical Center, Chicago, IL; Loyola University Medical Center, Maywood, IL; Loyola University Medical Center, Lombard, Illinois

## Abstract

**Introduction:**

A patients’ perception of their abilities to complete daily tasks post-burn injury can be difficult to measure and warrants more research. To ensure patients with an upper extremity (UE) burn injury are gaining the functional outcomes they desire, one burn center developed a quality improvement (QI) project focused on burn patients’ perceived ability to complete daily tasks.

**Methods:**

Occupational Therapists (OTs) assessed burn patients’ perceived UE function at evaluation and discharge with the QuickDASH, a self-reported outcome measure. Scores range from 0-100, higher scores indicate greater perceived deficits. The minimal clinically importance difference (MCID) is 15.9. Inclusion criteria was at least 18 years old, UE burn injury, hospitalized for > 48 hours, and cognitively able to participate. Data was collected & analyzed using Microsoft Excel. Normative data including means and standard deviations were reported. An analysis of variance (ANOVA) was performed to identify if changes in QuickDASH scores were significantly different for operative and non-operative patients.

**Results:**

115 patients met inclusion criteria from Sept. 2022 - Sept. 2023. 70.1% patients were male, 29.9% were female. The average total body surface area burned was 9.8%. The ANOVA did not find a significant difference in changes in QuickDASH scores between operative and non-operative patients (p=.79). The number of patients from initial data collection period to most recent who met the inclusion criteria increased, of those, the amount that completed a QuickDASH upon evaluation and discharged increased. See Table 1 below for the three data collection periods.

**Conclusions:**

The QuickDASH is a helpful tool for OTs working in the acute phases of burn rehabilitation. Tracking QuickDASH scores for patients with an UE burn injury contributes to continuous efforts of improving the quality of care and promoting patients’ achievement of desired outcomes. The QuickDASH can identify therapy needs beyond an acute care setting and promotes patient engagement.

**Applicability of Research to Practice:**

This QI project promotes continued growth for the burn center and adds to the body of knowledge about acute phases of burn rehabilitation. An area of opportunity for a future QI project includes implementing the QuickDASH in an outpatient setting, which would allow comparison to the patient’s acute care discharge score to promote continued progression towards desired outcomes. The presenters will continue to collect and analyze data prior to presentation if accepted.